# Bony Hyperostosis Recurrence after Complete Resection of Sphenoorbital Meningioma

**DOI:** 10.7759/cureus.1540

**Published:** 2017-08-04

**Authors:** Gmaan Alzhrani, William Couldwell

**Affiliations:** 1 Department of Neurosurgery, Clinical Neurosciences Center, University of Utah; 2 Department of Neurosurgery, University of Utah School of Medicine; Huntsman Cancer Institute

**Keywords:** hyperostosis, meningioma, recurrence, skull base, sphenoorbital

## Abstract

Bony hyperostosis is commonly associated with meningioma growth and is considered one of the characteristic signs on imaging; however, recurrence of meningiomas in the sphenoorbital area, including associated hyperostosis, is typically precluded by gross total resection of the lesion. This 63-year-old man presented with progressive double vision and proptosis in the right eye. He underwent frontotemporal craniotomy and partial removal after magnetic resonance imaging demonstrated a right sphenoorbital meningioma extending to the orbit and middle fossa. He had transient improvement of his symptoms postoperatively but experienced a progressive recurrence of symptoms and new onset of right facial hypoesthesia in the distribution of V1 and V2. We performed a right frontotemporal craniotomy with removal of the nodular part, as well as extensive drilling. Although the postoperative computed tomography scan revealed a gross total resection, the five-year follow-up scan demonstrated a recurrent hyperostosis in the region of the lesser and greater sphenoid wings, the middle cranial fossa floor with inferior extension toward the infratemporal fossa, and the sphenoid sinus wall. After another redo surgery, the patient continues to be monitored with yearly imaging. The extent of surgical resection is one of the most important predictors of meningioma recurrence postoperatively, and cases of recurrence after gross total resection are rare.

## Introduction

The sphenoorbital area is the most common location for meningiomas in the skull base; these lesions represent 20% of all meningiomas and 4% of all orbital tumors [1-2]. Sphenoorbital meningioma, also known as “meningioma en plaque of the sphenoid wing,” usually arises from the lesser wing of the sphenoid bone, is often associated with bony hyperostosis, and may invade the frontal, middle cranial base, orbit, and nasal sinuses [3-4]. Rates of recurrence for sphenoorbital meningioma depend on the extent of surgical removal, and some authors advocate an aggressive surgical resection for these tumors, including the hyperostotic bone and dural attachments, to limit recurrences. In this report, we discuss a rare case of a bony recurrence after the complete removal of a World Health Organization (WHO) Grade I sphenoorbital meningioma and discuss the relevant literature.

## Case presentation

**Figure 1:**
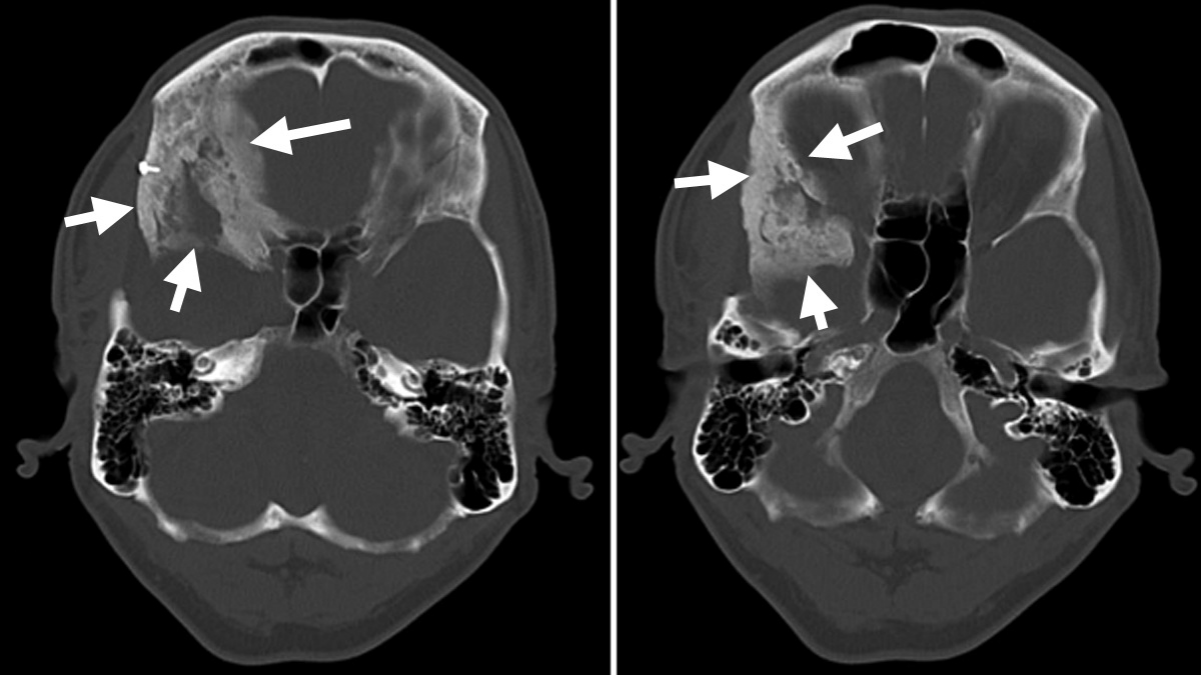
Preoperative computed tomography scan bone window images Axial cuts showing extensive hyperostosis of the lesser and greater sphenoid wing, lateral orbital wall, clinoid, and middle cranial fossa floor (arrows).

A 63-year-old man presented to an outside institution with a history of progressive double vision and proptosis in the right eye over a three-month period. On examination, he had an afferent pupillary defect in the right eye, 7 mm of exophthalmos, and an inferior visual field defect in the right eye. Magnetic resonance imaging (MRI) of the brain revealed right sphenoorbital meningioma extending to the orbit and middle fossa for which he underwent a right frontotemporal craniotomy and partial removal. The pathological diagnosis was WHO Grade I meningioma. The patient had transient improvement of his symptoms postoperatively but experienced a progressive recurrence of his symptoms and a new onset of right facial hypoesthesia at the distribution of V1 and V2. Computed tomography (CT) and MRI scans of the brain showed recurrence of his meningioma with extensive hyperostosis of the lesser sphenoid wing, the lateral and posterior wall of the orbit, and the middle fossa (Figure 1). The patient was referred to our institution for further management.

**Figure 2:**
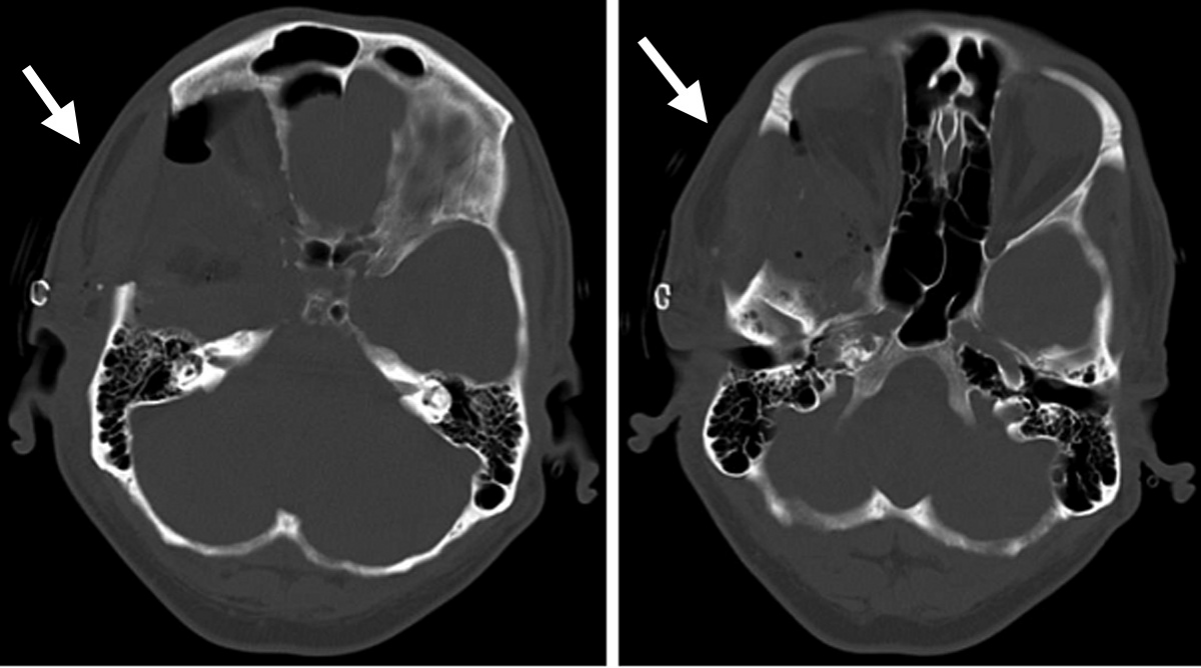
Postoperative computed tomography scan bone window images Axial cuts showing complete removal of the sphenoid bony hyperostosis, lateral and superior orbital wall, clinoid, and floor of the middle cranial fossa (arrows).

We performed a redo right frontotemporal craniotomy with removal of the nodular part, as well as extensive drilling of the lesser sphenoid wing, sphenoid ridge, lateral orbital wall, and clinoid, and decompression of the optic nerve, superior orbital fissure, foramen rotundum, and cavernous sinus lateral wall. A postoperative CT scan showed a gross total removal of the lesion (Figure 2), and the pathological specimen showed recurrent Grade I meningioma. The patient’s presenting symptoms and physical findings improved markedly.

**Figure 3:**
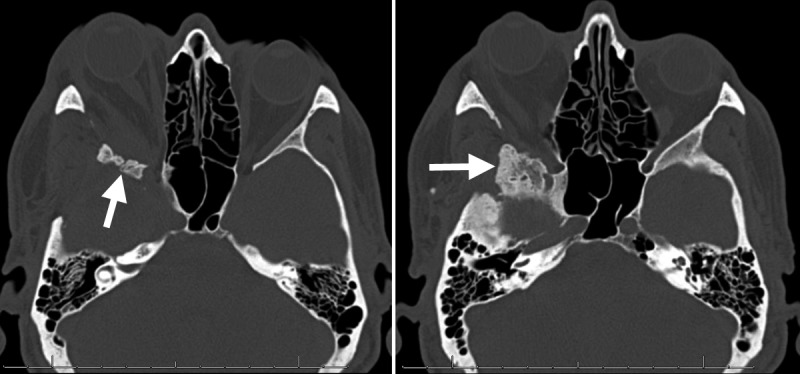
Computed tomography of the orbit Axial cuts showing recurrent bony growth of the sphenoid wings, lateral sphenoid sinus wall, and middle cranial fossa floor (arrows).

After five years of continued yearly imaging follow-up, the patient’s CT scan revealed a recurrent hyperostosis in the region of the lesser and greater sphenoid wings, middle cranial fossa floor with inferior extension toward the infratemporal fossa, and sphenoid sinus wall (Figure 3). The patient continues to be monitored with yearly imaging.

## Discussion

The recurrence in our case is purely bony hyperostosis without a soft tissue component on imaging and, so far, has not required surgical removal because the patient is asymptomatic. Depending on the extent of the tumor on imaging and patient presentation, in our practice, the goals of surgery for sphenoorbital meningioma include the removal of the tumor mass, decompressing the optic nerve, superior orbital fissure, and the orbital contents. These goals are achieved by removing the adjacent dura with a safe margin when possible, drilling the orbital roof and lateral wall, completing an anterior clinoidectomy, decompressing the optic nerve and superior orbital fissure, and removing any intraorbital tumor extension along with the periorbita. The bony removal in these cases typically extends as far medially as the posterior ethmoid sinus and lateral wall of the cavernous sinus and as far laterally as the foramen rotundum and maxillary division of the trigeminal nerve. When the sinus wall is violated, a muscle plug with fibrin glue is used to prevent a cerebrospinal fluid leak. In our experience, removal of all tumor, including soft tissue and hyperostotic bone, provides the longest recurrence-free survival and symptomatic relief for patients.

To our knowledge, this is the first report of a recurrent hyperostosis after a gross total resection of sphenoorbital meningioma with complete removal and drilling of the involved orbital bone, sphenoid bone, and middle cranial fossa floor. Sphenoorbital meningiomas can be primarily intraosseous without nodular growth intra- or extradurally [5]. Hyperostosis of the adjacent bone is thought to be due to preceding trauma, vascular hyperperfusion of the bone, irritation of the bone without invasion, stimulation of osteoblasts by factors secreted by the tumor cells, production of bone by the tumor itself, or tumor invasion of the bone [6]. Histological study of hyperostosis in large meningioma series has shown meningiomatous infiltration of the bone, and the authors advocate complete removal of hyperostotic bone when possible to reduce the risk of recurrence [1, 6].

The extent of surgical resection of sphenoorbital meningioma is a very important determinant of postoperative recurrence. Recurrence rates in recent studies have typically been between 5 and 10%. In one series of 25 patients with sphenoorbital meningioma, 70% of patients underwent complete surgical resection, and the five-year recurrence rate was 8% [7]. In another series of 67 patients, 82.3% had gross total removal, and the recurrence rate over 36 months was 7.1% [8]. In the third series of 33 patients, 6% of patients had a recurrence of the tumor over 4.5 years [3]. A higher recurrence rate of 29.7% was reported in a series of 47 patients with sphenoorbital meningioma in which only 51% had gross total removal [9].

## Conclusions

Complete removal of hyperostosing sphenoorbital meningioma is often limited by the complexity of skull base approaches, the proximity of the cranial nerves, poor accessibility, dural attachments, and an involvement of structures in the extracranial compartment, especially the nasal sinuses. However, whenever possible, gross total removal of these tumors, including the dura and surrounding bone, should be one of the goals of the surgery, especially in young and healthy patients to prevent recurrence.

## References

[1] Bowers CA, Sorour M, Patel BC, Couldwell WT (2016). Outcomes after surgical treatment of meningioma-associated proptosis. J Neurosurg.

[2] Bonavolonta G, Strianese D, Grassi P, Comune C, Tranfa F, Uccello G, Iuliano A (2013). An analysis of 2,480 space-occupying lesions of the orbit from. Ophthal Plast Reconstr Surg.

[3] Sughrue ME, Rutkowski MJ, Chen CJ, Shangari G, Kane AJ, Parsa AT, Berger MS, McDermott MW (2013). Modern surgical outcomes following surgery for sphenoid wing meningiomas. J Neurosurg.

[4] Nakamura M, Roser F, Jacobs C, Vorkapic P, Samii M (2006). Medial sphenoid wing meningiomas: clinical outcome and recurrence rate. Neurosurgery.

[5] Elder JB, Atkinson R, Zee CS, Chen TC (2007). Primary intraosseous meningioma. Neurosurg Focus.

[6] Pieper DR, Al-Mefty O, Hanada Y, Buechner D (1999). Hyperostosis associated with meningioma of the cranial base: secondary changes or tumor invasion. Neurosurgery.

[7] Shrivastava RK, Sen C, Costantino PD, Della Rocca R (2005). Sphenoorbital meningiomas: surgical limitations and lessons learned in their long-term management. J Neurosurg.

[8] Bikmaz K, Mrak R, Al-Mefty O (2007). Management of bone-invasive, hyperostotic sphenoid wing meningiomas. J Neurosurg.

[9] Talacchi A, De Carlo A, D'Agostino A, Nocini P (2014). Surgical management of ocular symptoms in spheno-orbital meningiomas. Is orbital reconstruction really necessary?. Neurosurg Rev.

